# Evaluating the effectiveness of a new student-centred laboratory training strategy in clinical biochemistry teaching

**DOI:** 10.1186/s12909-023-04272-7

**Published:** 2023-05-27

**Authors:** Guoying Xu, Chuanxiang Zhao, Mengdan Yan, Xiaoxian Zhang, Ling Zhu, Jiaxiu Liu, Yaping Zhao, Yuling Zhang, Weili Cai, Hongxiang Xie, Yuzhang Jiang, Qixiang Shao

**Affiliations:** 1 Division of Laboratory Medicine, School of Medical Science and Laboratory Medicine, Jiangsu College of Nursing, Huai’an, Jiangsu 223305 P. R. China; 2 Youyang Medical Laboratory Co., Ltd, Changzhou, Jiangsu 213200 P. R. China; 3 Laboratory Medicine Center, Department of Clinical Laboratory, Zhejiang Provincial People’s Hospital, Affiliated People’s Hospital, Hangzhou Medical College, Hangzhou, Zhejiang 310014 P. R. China; 4 Department of Medical Laboratory, Huai’an First People’s Hospital, The Affiliated Huai’an No.1 People’s Hospital of Nanjing Medical University, Huai’an, Jiangsu 223300 P. R. China

**Keywords:** Student-centred, Case-based learning, Clinical biochemistry, Laboratory training

## Abstract

**Background:**

The error-proneness in the preanalytical and postanalytical stages is higher than that in the analytical stage of the total testing process. However, preanalytical and postanalytical quality management has not received enough attention in medical laboratory education and tests in clinical biochemistry courses.

**Methods/approach:**

Clinical biochemistry teaching program aim to improve students’ awareness and ability of quality management according to international organization for standardization 15,189 requirements. We designed a student-centred laboratory training program, according to case-based learning that included 4 stages: “establish an overall testing process based on the patient’s clinical indicator, clarify principles, improve operational skills, and review process and continuous improvement”. The program was implemented in our college during the winter semesters of 2019 and 2020. A total of 185 undergraduate students majoring in medical laboratory science participated in the program as a test group, and the other 172 students were set up as the control group and adopted the conventional method. The participants were asked to finish an online survey to evaluate the class at the end.

**Results/outcomes:**

The test group had significantly better examination scores not only in experimental operational skills (89.27 ± 7.16 vs. 77.51 ± 4.72, *p* < 0.05 in 2019 grade, 90.31 ± 5.35 vs. 72.87 ± 8.41 in 2020 grade) but also in total examination (83.47 ± 6.16 vs. 68.90 ± 5.86 in 2019 grade, 82.42 ± 5.72 vs. 69.55 ± 7.54 in 2020 grade) than the control group. The results of the questionnaire survey revealed that the students in the test group better achieved classroom goals than those in the control group (all *p* < 0.05).

**Conclusions:**

The new student-centred laboratory training program based on case-based learning in clinical biochemistry is an effective and acceptable strategy compared with the conventional training program.

## Introduction/background

Clinical biochemistry is a pivotal division of the medical laboratory. According to the International Federation of Clinical Chemistry (IFCC), clinical chemistry is responsible for applying chemical, molecular and cellular strategies and techniques to better understand, and assess human health and disease processes. It ultimately affects the process of treatment as well as the quality of medical achievement [1]. It has been reported that the results of laboratory tests influence 70% of medical diagnoses, guide approximately 70% of clinical decisions and facilitate the provision of optimal patient care [2, 3]. Practical training plays a crucial role in clinical biochemistry curriculum. The goal of the course is to enable students to remember the test procedure and understand the principle and medical significance, especially to ensure the accuracy of the test results. However, in traditional teaching, the quality control in the analytical process has received more attention, neglecting the quality control in the preanalytical and postanalytical processes in the experimental courses of clinical biochemistry teaching. In fact, the error-proneness in the analytical process is lower than that in preanalytical and postanalytical processes of the TTP [4]. Moreover, the quality management ability and awareness are much more important for students. The International Organization for Standardization 15,189 (ISO 15189): Medical laboratories — requirements for quality and competence was first published by the ISO medical clinical laboratory and in vitro diagnostic system technical committee (ISO/TC212) in 2003. Now it has become an important international gold standard in medical laboratory proficiency cultivating and quality management after several revisions [5, 6]. They address the need to define and document processes and procedures throughout the TTP. Therefore, it is necessary for students to have quality management awareness by applying the ISO 15189 to the class.

Traditional training models such as lecture-based learning (LBL) have several features, including a teacher-centred tiered process, a focus on knowledge acquisition, and a final summative assessment at the end of courses. This is indeed the most cost-effective way to carry out theoretical education [7]. Whereas, several teaching modes are obviously superior to traditional teaching in the course of clinical biochemistry, such as traditional teaching combined with group discussion, peer debriefing approaches, and team learning [8–10]. However, small groups and case-based learning (CBL) are likely to dominate medical education. CBL is a learner-centred special type of problem-based learning (PBL) that guides students’ learning and exploration through cases. It has been elucidated that CBL can improve the performance and clinical skills of medical students [11]; help convey an understanding of key concepts [12]; improve clinical practice, problem-solving, case analysis and the link between theory and practice [13–15]; and motivate students to learn more deeply [16], with better student satisfaction [17]. It is hypothesized that students who participate in CBL gain deeper and longer lasting knowledge than those who do not [18]. Compared with traditional methods, the applying practical knowledge (Objective Structure Clinic Examination, OSCE scores) of CBL is significantly improved [19]. A limitation of this approach is that multiple faculty facilitators may be needed. During the COVID-19 pandemic, virtual teaching workshops have emerged as an easy and straightforward way to implant a more interactive format into virtual case teaching for health professions [20]. However, there is no proper teaching model to improve the entire quality management process according to ISO15189 in clinical biochemistry courses.

Here, we designed a new student-centred training program based on CBL in the experimental teaching of a clinical biochemistry course. The purpose is to improve the ability and awareness of quality management of students majoring in medical laboratories.

## Methods

### Participant

A total of 357 undergraduate students majoring in medical laboratories in 2019 and 2020 were randomly divided into two groups: the test group and the control group. Students participated the program each semester. There were 92 recruited into the testing group in 2019 and 93 recruited into 2020 according to individual will. The number of male and female students was kept similar to exclude the influencing factors of sex on CBL [13]. The remaining students (87 in 2019, 85 in 2020) participated in the traditional program were set up as a control group. Teachers with ≥1 year of CBL teaching experience are designated as the teachers of the test group, enrolling 10-12 students per training classroom. All study participants completed basic medical courses related to the testing profession and had a certain ability to comprehensively analyse medical knowledge. Students from both groups are taught by the same teachers using the same syllabus and teaching materials. In this study, no significant differences were found between the study participants, such as the theoretical score of biochemistry and clinical disease synopsis course. The control group was given appropriate supplementary training after the examination to prevent the unfair of education. All the programs were approved by education committee of our college.

### Teaching strategies

A total of 9 experiments were assigned. One subject is biosafety and the usage of biochemical instruments commonly used in clinical practice. The themes of the remaining 8 classes are specific experiments on clinical indicators of diabetes mellitus, liver cirrhosis, nephrotic syndrome, coronary atherosclerotic cardiopathy, pancreatitis, electrolyte disturbance, multiple myeloma, and hyperthyroidism. At the end of program, the lab examination was performed. Each experiment was conducted in three consecutive classes of 45 min. A similar learning environment was maintained for both groups –, i.e., lab classrooms, lecture times, assessment methods.

#### Test group

The laboratory training adopts a new student-centred training program that divided into 4 stages. First, students had access to the case (with the questions) at least 2 - 4 days before the class and were asked to answer several basic questions individually about the case before the class (What is the diagnosis based on? What are the detection indicators? What are the procedures for pre-analysis, analysis and post-analysis the indicators of a certain inspection?). The answers of each minor group were then shared in the class, and the students tried to reach a consensus among the groups, with the teachers’ facilitation. This stage took approximately 30 min. Second, it took 30 min to learn principles, which was mainly an explanation of the current commonly used methods and principles. Third, it took 45 min to improve their lab skills, including the evaluation of lab conditions, assessment of equipment conditions, use of internal control, and sample processing according to the standard operating procedure (SOP). Fourth, results were analysed by combining the ISO15189 requirements with the teaching contents to improve the operations in 30 min. The main concern was the review and reporting of results. When abnormal or suspicious results occurred, the students were able to identify them. The teachers facilitated the entire process. If the results were not judged correctly, the teacher asked students to re-check the result until they met the re-inspection requirements, and the students analysed whether the results could be issued. After that, the students were asked to conduct a quiz and an after-class survey.

#### Control group

The knowledge and theoretical outline of clinical biochemistry course in the lectures was the same as that of the test group. Experimental teaching was implemented in a teacher-centred way. The teacher explained the principles, operation points, and medical significance, and then the students performed the experiment.

**Fig. 1:**
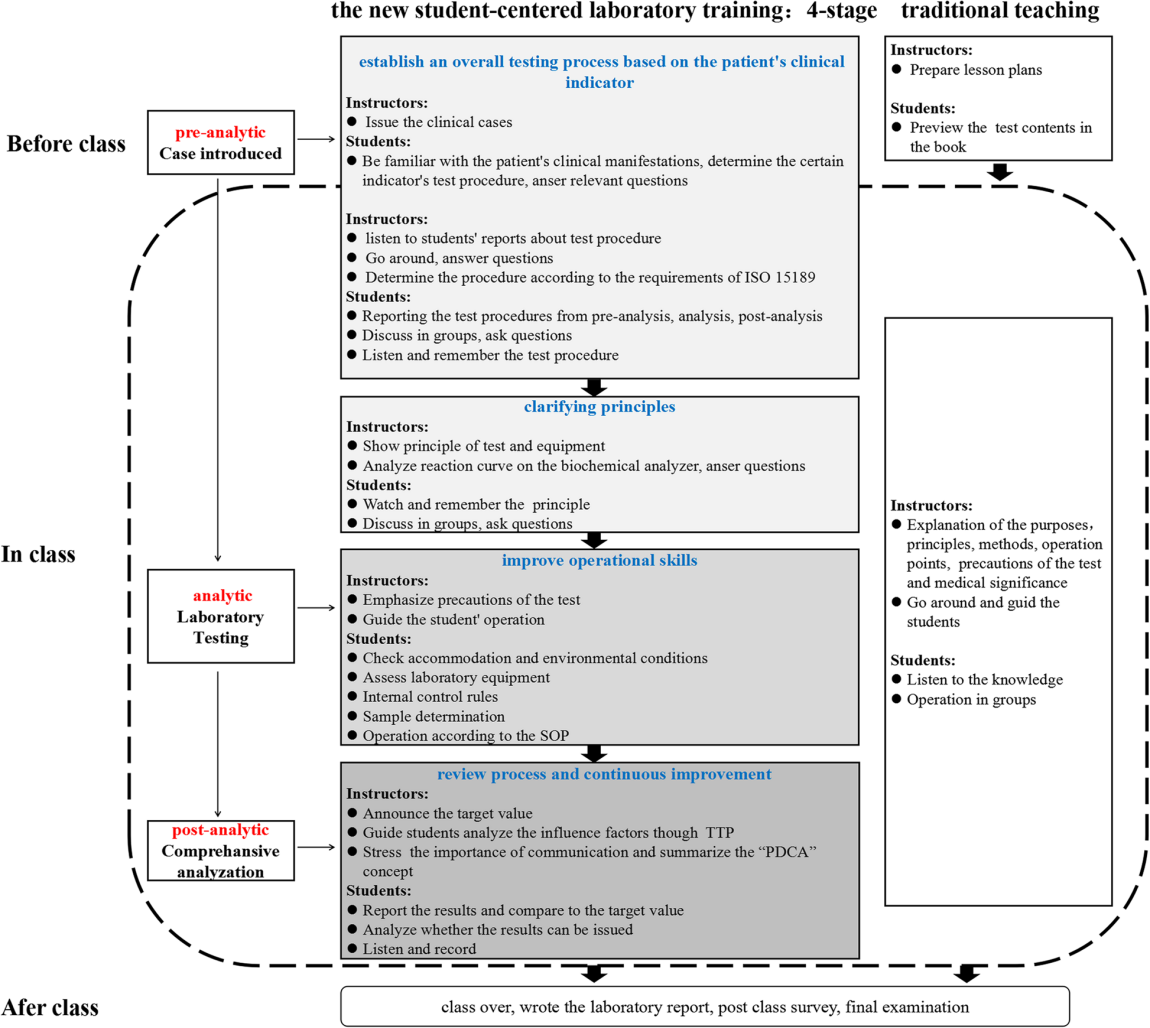
Schematic diagram of the pathway comparison between the new student-centred laboratory training program and the traditional training program in clinical biochemistry. SOP: Standard Operating Procedure; TTP: Total Testing Process; PDCA: Plan, Do, Check, Act

A schematic diagram of the teaching mode between the two groups (Fig. 1).

Give an example of the first stage: students were familiar with the clinical manifestations of the patient with recurrent systemic edema (finally diagnosed as nephrotic syndrome) 2 weeks before the class. When the nephrotic syndrome is diagnosed, urinalysis, blood counts and coagulation panel, renal function and electrolytes, liver panel, glucose tests were required to perform. Students were asked to report why and how the test procedure for “creatine and urea” in renal function was determined. And then students will be asked to discuss the examination process and medical significance, analyse various factors that may affect the test result, including pre-pre-analysis (test selection, test ordering, patient/specimen identification), pre-analysis (specimen collection, transportation, specimen processing, specimen preparation), analytic, post-analysis (report review, result reporting) and post-post-analysis (result interpretation) in accordance with the requirements of ISO 15189 quality system [21]. Then students make an operation plan according to the inspection process of the project, and the teachers evaluate and determine the testing procedure.

### Outcome evaluation

#### Assessment for laboratory operation

To evaluate the students’ ability and awareness of quality management in TTP and laboratory skills, the evaluation indicators were designed as shown in Table 1.

**Table 1:** The evaluation system of the experimental operation

Phase		Detailed Rules of Evaluation Index	Grade Weights and Within area	Percentages of Total	Evaluation Mode
1	Preanalytical	• Familiarity with the clinical significance	10%	40%	Oral test
		• Factors influencing the test procedure	10%		Oral test
		• Principle of test indicator	10%		Paper test
		• Parameter setting according to the instructions of the kit	10%		Observation
2	Analytical	• Check accommodation and environmental conditions, assess laboratory equipment	5%	40%	Observation
		• Use of internal quality control rules	5%		Oral test
		• Sample determination	10%		Oral test
		• Sample and reagent addition using the pipettes and micro-pipettors	10%		Observation
		• Records to be legible	5%		Observation
		• Instrument maintenance	5%		Observation
3	Postanalytical	• Results reporting	5%	20%	Paper test
		• Results analysis and judgement: evaluate them in conformity with clinical information available regarding patient	10%		Paper test
		• Communication with doctors	5%		Oral test

#### Assessment of students’ course scores

The total course scores included four components: classroom performance, experimental evaluation, middle and final examination (Table 2). Classroom performance includes attendance, attitude, completeness of assignment, and experiment report. The content of the experiment report includes 4 parts: how to determine the test procedure, test principle, precautions of the procedure, and results interpretation and analysis. The experimental evaluation was carried out in the last class. The eight items were numbered, and the students drew lots to determine which items to evaluate. The mid-term and final exams had terminology, short answers and single-choice questions: 10 fill-in-the-blank (1 point per question), 5 terminology (two points per question), 4 short-answer questions including one case analysis (5 points per question) and 60 single-choice (1 point per question). Standard answers to all questions were defined by the instructor before the students’ answers were graded.

**Table 2:** Composition of clinical biochemistry course grades

Component	Specific Definition	Percentages
Usual performance	attendance, attitude, completion of assignment, experimental report, class participation online and offline	30%
The laboratory evaluation	score using experimental evaluation system	20%
Mid-term examination	written examination	20%
Final exam	written examination	30%

#### Questionnaire survey

Curriculum evaluation is critical to continuous assurance of teaching quality [22]. To assess the effectiveness and acceptability of implementing the 4-stage experimental training program based on the ISO 15189, in addition to the typical course evaluations, the students were asked to complete a survey about the course after finishing the course. An anonymous 10-question survey was created to develop a baseline of student achievement of goals in the class and the impact of teaching mode on learning (Table 3). The questions presented in the results section were discussed by all supervisors involved in this study to ensure their quality. Most of the survey questions were in Likert scale format, giving a statement on a scroll bar that the students could choose from “Strongly Disagree” to “Strongly Agree” on a scale of 1-5.

**Table 3:** Questionnaire survey

Objectives for the class	
**1**	I remembered the test procedure.
**2**	I understand the principle of the test.
**3**	Clinical cases help me understand the medical significance of examination.
**4**	I deeply understand the meaning of ISO 15189 requirements.
**5**	I agree with the idea of “quality control is fundamental, quality management is the most important objective”.
Impact of teaching mode on learning	
**6**	I will pay more attention to the influencing factors test results in the preanalytical and postanalytical processes.
**7**	I will keep records of every step of the experiment.
**8**	I will communicate with clinical staff and patients actively after examination.
**9**	I am interested and satisfied with the teaching process designed.
**10**	I hope the 4-stage teaching model will be used in other courses.

### Statistical analysis

Means and standard deviations were calculated, and the differences were analysed using an independent samples *t* test. A *p* value < 0.05 was considered to be statistically significant. Data are presented as the means ± SDs.

## Results

### Comparison of laboratory training scores

A total of 357 students participated in this program, and 185 students (52%) attended a 4-stage training program. A total of 314 students completed the post-class survey (88% response rate).

**Fig. 2:**
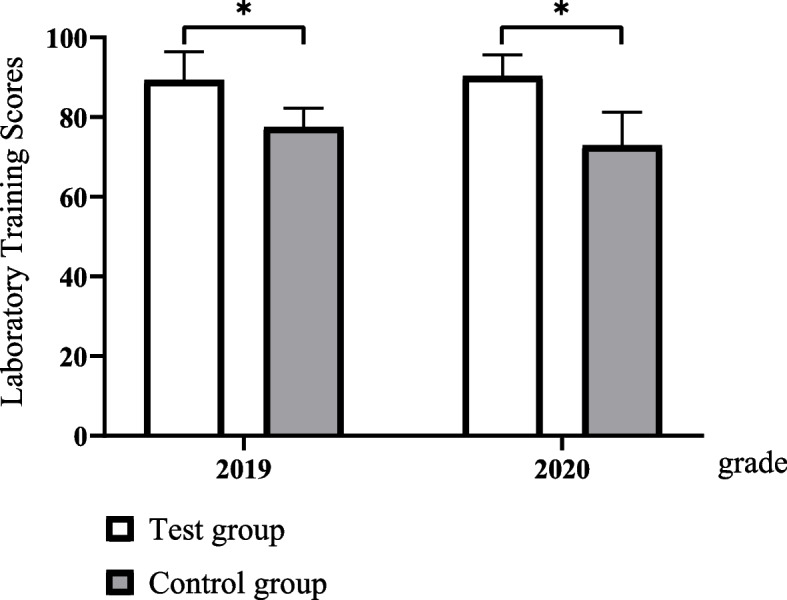
Comparison of laboratory training scores (^*^P < 0.05)

The experimental operation scores in 4-stage training program classes were significantly higher than those of the traditional program classes of both grades (Fig. 2).

### Comparison of total course scores

**Fig. 3:**
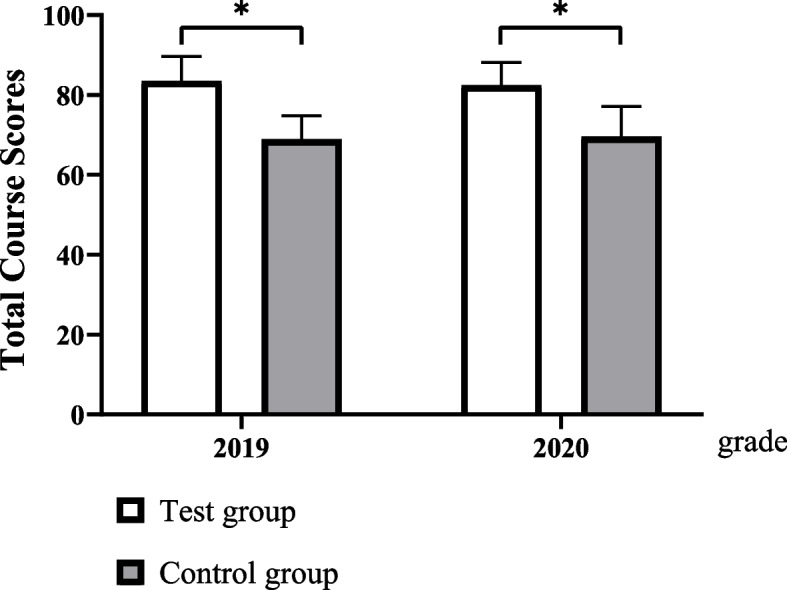
Comparison of total course scores (^*^P < 0.05)

The students’ scores for this course in the test groups were significantly higher than those in the control groups in 2019 and 2020 (Fig. 3).

### Survey results

The accumulative score on the survey questions on learning for the students in test group was higher than that for the students in control group either about objectives or the impact of teaching mode on learning (Table 4). Students in the testing group agreed that they remembered the testing procedure, understood the testing principle and the medical significance, understood the connotation of ISO 15189 requirements, and agreed that quality management is more important than quality control. Fewer students in the control group agreed. More students were interested and satisfied with this new student-centred teaching mode. Meanwhile, more students would like the teaching model to be used in other courses.

**Table 4:** Questionnaire survey comparison

Survey Objectives	Aggregate score		*t* value	*P* value
	Test group (*n* = 160)	Control group (*n* = 154)		
Achievement of curriculum objectives	22.64 ± 2.58	18.31 ± 3.97	11.41	< 0.05
Impact of teaching mode on learning	23.21 ± 2.04	19.77 ± 3.69	10.17	< 0.05

## Discussion

Clinical biochemistry belongs to the field of medical technology and involves various techniques and methods for analysing the chemical components of body fluid samples [23]. To do well in the clinical biochemistry course, students will undergo rigorous clinical laboratory practical training and gain valuable practical experience in sample handling and instrumentation. Ensuring the accuracy of test results and understanding the medical significance of laboratory tests are important for students who major in medical laboratories. There are many obstacles to teaching clinical chemistry and laboratory medicine, such as the lack of interactive or hands-on teaching models [24]. In this study, we designed a new student-centred training program with 4 stages based on CBL considering TTP, including preanalytical, analytical, and postanalytical stages. The participating teachers were asked to provide clinical cases related to clinical biochemistry, design questions according to the experimental objectives, and upload to the DingTalk group before class. Reaction curves on an automated biochemical analyser were also provided. CBL requires students to prepare well in advance, which may be considered an additional burden if they are not yet familiar with the subject of the course [25, 26]. The authors suggest that CBL should be actively adopted for courses that are delivered in the final stage of the program. The quality of students’ previews directly affects the learning effect of each stage. In particular, the determination of the process of the first stage of the inspection program process and the analysis of errors in the final stage from pre-analysis to post-analysis.

Our new student-centred training strategy has a positive effect on both the achievement of class goals and the impact of teaching mode on learning. It has several advantages over traditional teaching methods. First, it helps students combine the theory with complicated clinical situations. With cases as a bridge, to explore as a driving force, so that students can integrate their knowledge and adapt to clinical practice. Second, under the new training evaluation system, students pay more attention to the management of the entire testing process rather than on the quality control of the analysis, as the error rate of the analysis is lower than that of the pre-analysis and post-analysis of the TTP. Third, by analysing the experimental results and unconsciously recording the test process, ISO 15189 concepts of continuous improvement were implanted, thereby putting the concept of Plan Do Check Act (PDCA) into practice, developing recording habits, and improving communication skills.

While the program has already produced very positive results, there are many improvements and additions that could be made. The first would be to check students’ familiarity with the case before the class. Otherwise, the first stage of the study time could be extended. In addition, multiple discipline inspection items, such as immunology, microbiology, and clinical examination, should be considered based on the symptoms of each patient. Different assignments of students may affect the course sores between the two groups.

## Conclusion

In summary, our experience suggests that this new student-centred experimental teaching strategy based on CBL is more effective and acceptable than the conventional experimental teaching mode in the clinical biochemistry course.

## Data Availability

The datasets used and/or analysed in this study are available from the corresponding authors on reasonable request.

## Abbreviations

ISO 15189: International Organization for Standardization 15,189
IFCC: International federation of clinical chemistry
TTP: Total testing process
LBL: Lecture-based learning
CBL: Case-based learning
PBL: Problem-based learning
OSCE: Objective structure clinic examination
SOP: Standard operating procedure
PDCA: Plan, Do, Check, Act
